# Occlusal traits and orthodontic treatment need in 7‐ to 10‐year‐olds in Estonia

**DOI:** 10.1002/cre2.64

**Published:** 2017-04-26

**Authors:** Hettel Sepp, Mare Saag, Anna‐Liisa Svedström‐Oristo, Timo Peltomäki, Heli Vinkka‐Puhakka

**Affiliations:** ^1^ Department of Stomatology University of Tartu Estonia; ^2^ Department of Oral Development and Orthodontics, Institute of Dentistry University of Turku Finland; ^3^ Faculty of Medicine and Life Sciences University of Tampere Finland

**Keywords:** dental occlusion, ICON, treatment need

## Abstract

The aim of this study was to evaluate the distribution of occlusal traits and orthodontic treatment need and complexity in Estonian 7‐ to 10‐year‐old children. This data provides solid information for planning of orthodontic care. Data of 392 Estonian children (198 girls and 194 boys, mean age 9.0 years, range 7.1–10.4 years) was analysed in this cross‐sectional study. Assessed traits included first molar and canine sagittal relationship, overjet, overbite, crowding, midline diastema, crossbite, and scissor bite. Orthodontic treatment need and complexity were assessed using the Index of Complexity, Outcome, and Need. Parents' opinion regarding their child's teeth was determined using a questionnaire. The most prevalent occlusal traits were canine class I sagittal relationship (73.7%), midline diastema (73.0%), molar class I sagittal relationship (57.4%), and overbite ≥3.5 mm (51.8%). According to the Index of Complexity, Outcome, and Need, 64.3% of Estonian elementary school children were in need of orthodontic treatment. Treatment complexity was simple in 12.5%, mild in 38.8%, moderate in 22.7%, difficult in 14.0%, and very difficult in 12.0% of the children. Approximately 66.4% of the parents felt that their child needed orthodontic treatment. This study confirms earlier findings indicating that the most frequent sagittal relationship is class I in the first molars and class I in the canines. However, the sagittal relationship was asymmetric in more than half of the children. Correlation between objectively defined treatment need and parents' desire for treatment was high in Estonia.

## 1 INTRODUCTION

Orthodontic treatment has been found to improve oral health‐related quality of life (Silvola, Rusanen, Tolvanen, Pirttiniemi, & Lahti, 2012), and demand for orthodontic treatment is growing in many countries. Increased attention is paid to facial and dental appearance, which have been found to affect, for example, social relationships, self‐esteem, and conclusions others make on the basis of external characteristics (Kerosuo, Hausen, Laine, & Shaw, 1995; Nisbett & Wilson, 1977; Ritter, Casey, & Langlois, 1991). There are also rare associations between dental features and functions distant to the oral cavity (Giuca, Caputo, Nastasio, & Pasini, 2011; Hultcrantz & Löfstrand‐Tideström, 2009; Miles, Vig, Weyant, Forrest, & Rockette, 1996; Monaco et al., 2011; Schütz‐Fransson & Kurol, 2008).

In publically funded orthodontic care, data on occlusal traits among 7‐ to 10‐year‐olds are essential for planning of treatment strategies. Early mixed dentition stage provides a good time point to consider interceptive orthodontics (Kerosuo, Väkiparta, Nyström, & Heikinheimo, 2008; Sunnak, Johal, & Fleming, 2015). In the oldest children of this age group, clinical signs of skeletal and dental deviations are often clearly visible enabling the further planning of, for example, work force and division of work.

Planning of population‐based, cost‐effective health services should be founded on data focusing on the target country. Because no data exist on the prevalence of occlusal traits and orthodontic treatment need in Estonia, this study is the first in a series of extensive investigations analysing the prevalence of occlusal traits in children and adolescents from 3 to 21 years of age. Parents' and adolescents' expectations and perceptions concerning orthodontic treatment are also evaluated. The aims of this study were

- to evaluate the distribution of occlusal traits in 7‐ to 10‐year‐old children in Estonia and
- to evaluate the objective and subjective need for orthodontic treatment in Estonia.

## 2 METHODS

### 2.1 Subjects

Recruitment of 7‐ to 10‐year‐old children started in November 2009 and was completed in December 2010. A multistage stratified cluster sampling design was implemented. The recruitment took place in four randomly selected elementary schools: one in Tallinn (Northern Estonia), two in Tartu (South Estonia), and one in Pärnu (South‐West Estonia). All the second‐grade children in selected schools were invited to participate in the study.

**Figure 1:**
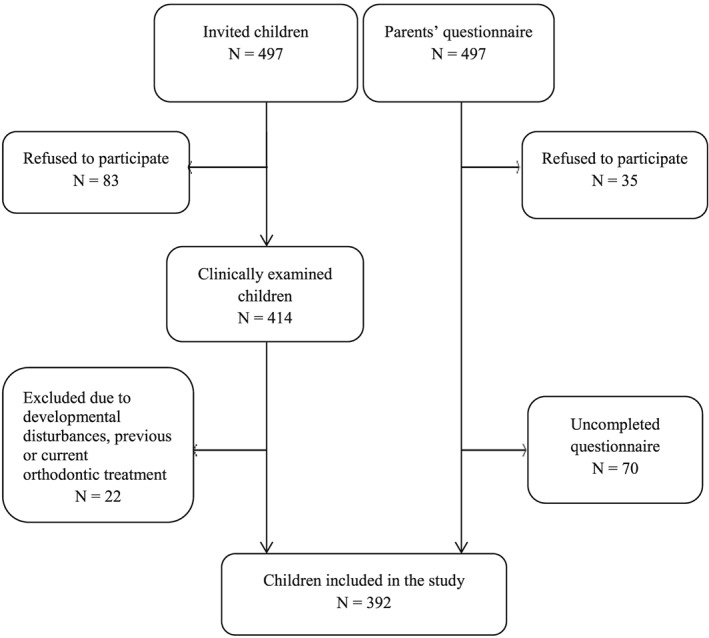
Selection of the final study sample

Sample size was determined with the aid of a statistical power calculation. The selection of children is illustrated in Figure 1. The final sample consisted of 392 children (198 girls and 194 boys, mean age 9.0 years, age range 7.1–10.4 years). Prior to the study, a written description of the study protocol was given to all children and their parents or guardians. All participants signed an informed consent form, indicating that their participation in the study was voluntary. The study protocol was approved by the Ethics Review Committee on Human Research of the University of Tartu (Protocol No. 186T‐24).

### 2.2 Clinical examination

Five occlusal traits were registered clinically in centric occlusion, separately for the left and right sides: (a) sagittal relationships of the first molars and canines, (b) overjet, (c) overbite, (d) crossbite, and (e) scissor bite. All clinical examinations were performed by Examiner 1. The examination was carried out in the school's dental office using a dental mirror, probe, pencil (0.3 mm), and millimetre ruler (Dentaurum 042‐751 Münchner Modell). Alginate impressions for plaster casts were taken from each child.

### 2.3 Plaster casts

Examiners 1 and 2 registered three features from the plaster casts in order to achieve consensus: (a) end‐to‐end relationship of the first molars and deciduous and permanent canines separately for the right and left side, (b) crowding, and (c) diastemas between central incisors. Registration of the occlusal traits was based on international standards (Brunelle, Bhat, & Lipton, 1996; Horowitz & Hixon, 1966; Moorrees, 1959); a detailed description of definitions is given in Appendix A. Furthermore, using the plaster casts, Index of Complexity, Outcome, and Need (ICON) for orthodontic treatment was assessed according to written instructions (Daniels & Richmond, 2000) by Examiner 1.

### 2.4 Reliability

Before the intended study after 1 week, Examiner 1 re‐examined clinically 22 children. To assure the validity of ICON assessments, 39 randomly selected plaster casts (10%) were analysed by both Examiner 1 and by an ICON calibrated Examiner 3.

The intraexaminer reliability was good (*r* > .97). The interexaminer reliability varied from moderate (*r* = .50) to good (*r* > .93).

### 2.5 Questionnaire

The guardians' opinions regarding a child's general dental health, tooth alignment, dental appearance, and opinion regarding orthodontic treatment need were gathered through five questions (Appendix B). The questionnaire was filled out at home by one of the parents. More than one answer per question was allowed. Questions were modified for this study from similar questions in a previous study (Pietilä & Pietilä, 1996).

### 2.6 Statistical analysis

The test–retest (Pearson's and Spearman's correlations) and chi‐square test were used to compare the frequencies of specific features (IBM SPSS v.20 software for Windows). *p* values of less than .05 were considered statistically significant.

## 3 RESULTS

### 3.1 Occlusal traits among 7‐ to 10‐year‐old Estonian children

The most prevalent occlusal traits in Estonian 7‐ to 10‐year‐old children were class I canine relationship (73.7%), midline diastema (73.0%), class I molar relationship (57.4%), and increased (≥3.5 mm) overbite (51.8%). The sagittal relationship in canines and molars was asymmetric in a large number of children (33.2% and 35.7%, respectively). The detailed prevalence of occlusal traits is presented in Table 1. There were only 17 children (4.3%, 9 girls and 8 boys) with symmetrical class I canine and molar relationships, overjet and overbite of 1–3 mm, without crowding, scissor bite, crossbite, and negative overbite. The overjet ranged from −1 to 10 mm (mean 3.1 mm, *SD* 1.67). The overbite ranged from −2 to 8.5 mm (mean 3.2 mm, *SD* 1.75). Crowding ranged from 1 to 8 mm in the maxillary and from 0.5 to 8.0 mm in the mandibular arch. The most frequent crowding was 2 mm in the maxillary and 1 mm in the mandibular arch (6.9% and 11.5%, respectively). Compared to boys, girls had 1.6 times greater odds of having crowding, and smaller odds of having canine end‐to‐end, overjet ≥3.5 mm, and class II molar relationship. The width of the midline diastema was most frequently 2 mm (20.2%) in the maxillary arch (range 0.5–4.5 mm) and 0.5 mm (range 0.5–4.5 mm) in the mandibular arch (6.1%).

**Table 1:** Prevalence of occlusal traits in 7‐ to 10‐year‐old Estonian children (*N* = 392)

	Occlusal trait	Prevalence (*N* = 392) %
Canine relationship	Class I	73.7
	Class II	3.6
	End to end	41.6
	Class III	2.3
	Symmetric	66.8
	Asymmetric	33.2
Molar relationship	Class I	57.4
	Class II	21.9
	End to end	54.1
	Class III	1.5
	Symmetric	64.3
	Asymmetric	35.7
Horizontal relationship	Overjet ≥3.5 mm	37.5
	Negative overjet	1.0
Vertical relationship	Overbite ≥3.5 mm	51.8
Transversal relationship	Posterior crossbite	10.2
	Scissor bite	1.5
Spacing	Midline diastema	73.0
	Maxillary	57.7
	Mandibular	15.3
Crowding	Upper and lower arch	49.7
	Maxillary	18.9
	Mandibular	37.9

### 3.2 Orthodontic treatment need and complexity of treatment

According to ICON, 64.3% of Estonian 7‐ to 10‐year‐olds were in need of orthodontic treatment (ICON score > 43). The scores ranged from 7 to 105 (median score 50), the most frequent score being 44 (11.2%). There were no gender differences in treatment need (*χ* ^2^(1) = 1.7, *p* = .21). Distribution of treatment complexity is presented in Table 2.

**Table 2:** Distribution of orthodontic treatment complexity in 7‐ to 10‐year‐old Estonian children determined with ICON

Complexity	ICON scores	Girls (*N* = 198)		Boys (*N* = 194)		Total (*N* = 392)	
		*N*	%	*N*	%	*N*	%
Simple and mild	<50	112	56.6	89	45.9	201	51.3
Moderate	51–63	40	20.2	49	25.3	89	22.7
Difficult	64–77	28	14.1	27	13.9	55	14.0
Very difficult	>77	18	9.1	29	14.9	47	12.0

*Note*. ICON = index of complexity, outcome, and need.

### 3.3 Parents' views and orthodontic treatment need

Approximately 65.6% of the parents were satisfied or very satisfied with their child's dental health. The association between parents' satisfaction and objective treatment need was statistically significant (*χ* ^2^(3) = 12.83, *p* = .005). More than half of the parents (53.1%) were satisfied or very satisfied with the alignment and appearance of their child's teeth. Satisfaction with the alignment of a child's teeth and parents' assessment of orthodontic treatment need were statistically significantly associated (*χ* ^2^(4) = 19.78, *p* < .001). Approximately 66.4% of parents felt that their child needed orthodontic treatment, mainly for improvement of dental appearance (37.1%), reduction in the amount of caries (23.1%), and ease of cleaning (19.6%). Approximately 56.5% of children whose parents did not want orthodontic treatment were in need of orthodontic treatment according to ICON (Table 3). Parents' opinion regarding treatment need did not differ for girls and boys. A statistically significant association was found between parents' desire to get a child's teeth straightened and treatment need as assessed using ICON (*χ* ^2^(1) = 5.59, *p* = .022).

**Table 3:** Parents' satisfaction with their child's dental health, and alignment of teeth, and their opinion on treatment need as compared to an assessment using ICON (7‐ to 10‐year‐old Estonian children; *N* = 392)

	Treatment need		Total	
	ICON ≤ 43 (*N*)	ICON > 43 (*N*)	*N*	%
Satisfaction with child's dental health				
Very satisfied	11	10	21	5.4
Satisfied	95	141	236	60.2
I don't care	0	1	1	0.3
Somewhat dissatisfied	30	78	108	27.6
Very unsatisfied	2	17	19	4.8
I don't know	0	1	1	0.3
No answer	2	4	6	1.5
Total	140	252	392	100.0
Satisfaction with the alignment of child's teeth				
Very satisfied	8	8	16	4.1
Satisfied	86	106	192	49.0
Somewhat dissatisfied	35	117	152	38.8
Very unsatisfied	3	3	6	1.5
I don't know	7	16	23	5.9
No answer	1	2	3	0.8
Total	140	252	392	100.0
Child needs orthodontic treatment				
Definitely not	2	3	5	1.3
No, I don't think so	45	58	103	26.3
Yes, I think so	63	145	208	53.1
Yes, definitely	17	35	52	13.3
No answer	13	11	24	6.2
Total	140	252	392	100.0

*Note*. ICON = index of complexity, outcome, and need.

## 4 DISCUSSION

This study is the first population‐based study registering occlusal traits and orthodontic treatment need among 7‐ to 10‐year‐olds in Estonia. The largest ethnic groups in Estonia are Estonians (68% of the population) and Russians (28%); the remaining 4.0% consist of 142 other ethnicities. For practical reasons, sampling was done in cities. Children were randomly selected and can be considered representative of those cities' child population. We have no reason to believe that the situation in the countryside or in other cities is different, because Estonia has neither big cities and slums nor long distances between population centres. Any possible regional differences in Estonia and their possible correlation to the socioeconomic status of families need to be assessed in future studies.

This study confirmed earlier findings, namely, that the most frequent sagittal relationship is class I in the first molars and class I in canines. Worth noting is that at the age of 7‐ to 10 years, asymmetric sagittal molar and canine relationships were frequent in Estonian children. Asymmetry or laterality (directional asymmetry) is a common finding in many craniofacial structures (Pirttiniemi & Kantomaa, 1992). Decay, early loss of the deciduous teeth, discrepancy in tooth size and quantity, derangements in the temporomandibular joint, or asymmetric habits may create asymmetric occlusal development (Moorrees, 1959; Thilander & Rönning, 1985). Prevalence of class II molar relationship was 21.9%, which is in line with the prevalence reported from other countries, 9.2–28.0% in mixed dentition (Dimberg, Lennartsson, Söderfeldt, & Bondemark, 2013; Lux, Dücker, Pritsch, Komposch, & Niekusch, 2009; Myllärniemi, 1970). In part, the variability may be related to differences in applied criteria and in the interpretation of findings. Most studies have not differentiated between sagittal symmetry and asymmetry. The range of overjet in 7‐ to 10‐year‐old Estonian children was similar to that of 9‐year‐old children in Germany (Lux et al., 2009), but unlike in Germany, there was no difference between boys and girls in Estonia. Compared with Finnish children, Estonian children had a higher prevalence of an increased overjet in mixed dentition (Myllärniemi, 1970). It is possible that Estonian children with an overjet of ≥6 mm would benefit from early orthodontic treatment (Artun & Al‐Azemi, 2009; Järvinen, 1978). The recent *Cochrane Review* also concludes that early orthodontic treatment is effective, for example, in reducing the incidence of incisal trauma (Thiruvenkatachari, Harrison, Worthington, & O'Brien, 2013).

The frequency of negative overbite was similar in Estonian and Finnish children but clearly lower than in a Swedish sample of 7‐year‐olds, where those with erupting incisors had already been excluded from analyses (Dimberg et al., 2013).

Despite the rarity of crossbite and scissor bite, they should be monitored for. Early treatment may prevent development of occlusal dysfunction and asymmetrical growth (Pirttiniemi & Kantomaa, 1992; Thilander, Wahlund, & Lennartsson, 1984).

In the age group of 7‐ to 10‐year‐olds, the youngest children were just entering to early mixed dentition, whereas some 10‐year‐olds already had permanent dentition. Hence, considerations for interseptive procedures range from assisting proper eruption of incisors and first molars to spacing or crowding and skeletal relationships. All dentists face these occlusal traits in yearly dental examinations. In addition, the frequent asymmetric sagittal relationships observed in this study bring additional challenge for orthodontic planning.

In this age group, there exists no generally accepted treatment need index, quick to apply, and use in assessment of plaster casts. Despite the fact that ICON is designed for late‐mixed dentitions (Daniels & Richmond, 2000), we used it for assessment of orthodontic treatment need. Its assessments are clearly determined and it also provides data on treatment complexity. The 10 included photographs Aesthetic Component of the Index of Orthodontic Treatment Need (AC/IOTN) represent slightly older children. However, they can easily be used as a reference to the level of dental aesthetics; there is no intention to point out a similar dentition to the one that the child has but to select the photograph with as pleasing an occlusion. As a matter of fact, there is no photograph representing, for example, negative overbite or class III relationship. In the future, the ICON scores will be comparable with those of the older age group of Estonian adolescents. Although ICON is useful in assessing treatment need, it does not assist in planning the timing of treatment, which is an important aspect of paediatric orthodontic care.

Girls seem to pay more attention to their appearance and are more interested in orthodontic treatment than boys (Birkeland, Bøe, & Wisth, 2000; Jung, 2010). In Estonia, parents' opinions were not influenced by the child's gender. Future studies should assess the age at which Estonian children themselves begin to pay attention to occlusal traits and dental appearance.

As determined with ICON, orthodontic treatment need in Estonia is higher than reported in epidemiological studies from Latvia and Finland (Heikinheimo, 1978; Liepa, Urtane, Richmond, & Dunstan, 2003). The expectations and wishes of parents are in line with ICON, partly because the index is sensitive to visible deviations. This study confirms earlier findings (Birkeland et al., 2000; Kiekens, Maltha, van't Hof, & Kuijpers‐Jagtman, 2006) that patients' main expectations regarding orthodontic treatment are related to improvement in dentofacial aesthetics. This study fills a significant knowledge gap in oral health in Estonia by providing an overview of dental traits among 7‐ to 10‐year‐old children.

## 5 CONCLUSION

The present results indicate that

- the most prevalent occlusal traits were class I canine sagittal relationship, midline diastema, class I molar sagittal relationship, and increased overbite;
- more than half of Estonian children had an asymmetrical canine and molar relationship;
- according to ICON, 64.3% of Estonian 7‐ to 10‐year‐olds were in need of orthodontic treatment;
- a statistically significant association was found between parents' desire to straighten their child's teeth and treatment need assessed using ICON. Parents' opinion regarding orthodontic treatment need did not differ in a statistically significant way on the basis of the child's gender; and
- the parents' main expectation from orthodontic treatment was improvement in dentofacial aesthetics.

## APPENDIX A Registration criteria of occlusal traits in the clinical examination and from the plaster casts

### A.1

#### A.1. Sagittal plane

The sagittal relationship of the first permanent molars was registered between perpendicular projections, on the occlusal plane, from the tip of the triangular ridge of the mesiobuccal cusp of the maxillary first permanent molar and the buccal groove of the mandibular first permanent molar.

*Molar class I:*: the triangular ridge articulated in the buccal groove of the mandibular first permanent molar.
*Molar class II:*: the triangular ridge articulated anterior to the mesial groove of the mandibular first permanent molar.
*Molar class III:*: the triangular ridge articulated posterior to the mesial groove of the mandibular first permanent molar.
*End to end:*: the triangular ridge of the mesiobuccal cusp of the maxillary first permanent molar articulated to the triangular ridge of the mesiobuccal cusp of the mandibular first permanent molar.

Molar classes II and III were registered in the accuracy of ≥1/2 cusp width. In cases of obvious tooth migration, no attempt was made to endeavour the original intercuspation. Registration was not done when the first molar was missing.

The sagittal relationship of the canines was measured between perpendicular projections, on the occlusal plane, from the tip of the maxillary canine and the contact point of the mandibular canine and the first deciduous molar or the first premolar.

*Canine class I:*: the tip of the maxillary canine occluded to the distal surface of the mandibular canine.
*Canine end to end:*: the tip of the maxillary canine articulated to the tip of mandibular canines. A deviation of 1 mm or more to the mesial or distal was classified as canine class II or I, respectively.
*Canine class III:*: the tip of the maxillary deciduous canine occluded more than 1 mm posterior to the distal surface of the mandibular canine

In the case of missing canine, the registration was not recorded. No attempt was made to compensate for drift of teeth due to premature extraction or any other reasons.

#### A.2. Horizontal plane

The overjet (positive) was measured in 0.5‐mm intervals as the horizontal distance, parallel to the occlusal plane, from the most labial surface of the lower central incisors to the most labial point of the incisal edge of the corresponding upper incisors. In case both upper or lower central incisors were missing, the lateral incisor was used.

A negative overjet was measured in 0.5‐mm intervals as the horizontal distance, parallel to the occlusal plane from the most labial surface of the upper central incisor to the most labial point of the incisal edge of the corresponding lower central incisors.

#### A.3. Vertical plane

The overbite (positive) was measured in 0.5‐mm intervals as the distance between the projection of the edge of the most overlapped central incisor on the labial surface of the lower incisors (in centric occlusion) and the incisal edge of the lower incisor.

A negative overbite or open bite was recorded when there existed a vertical space between the upper and lower incisal edges in the centric occlusion. The negative overbite was measured in 0.5 mm intervals from the incisal edge of the lower incisors to the incisal edge of the upper corresponding incisors.

#### A.4. Transversal plane

The posterior crossbite was registered in the canine, premolar, and molar area when the buccal cusp of the maxillary tooth occluded lingual to the buccal cusp of the mandibular antagonists (at least one pair of teeth). Teeth in an end‐to‐end position were registered as crossbite.

A scissor bite was recorded in the premolar and molar area when lingual cusps of maxillary teeth occluded buccally of the buccal surfaces of corresponding mandibular teeth.

#### A.5. Crowding

Crowding of the teeth was estimated as total space deficiency (in millimeters) of the anterior teeth (incisors only). The amount of crowding was recorded in the maxillary and mandibular arch as the difference between the total mesio‐distal tooth diameter and the arch circumference. The possible influence of growth in arch width was not estimated.

#### A.6. Midline diastema

Midline diastema between the central incisors in the upper and lower arch was measured in 0.5‐mm intervals between the mesial margin of the right and left incisors on the middle height of the tooth crown.

## APPENDIX B Questionnaire

### B.1

1. How satisfied are you with the dental health of your child?

Very satisfied/Satisfied/I don't care/Somewhat dissatisfied/Very unsatisfied/I don't know

2 How satisfied are you with the alignment of your child's teeth?

Very satisfied/Satisfied/Somewhat dissatisfied/Very unsatisfied/I don't know

3 Do you think that your child needs orthodontic treatment?

Definitely not/No, I don't think so/Yes, I think so/Yes, definitely

4 For what reason would you like to align your child's teeth?

To improve their appearance/To improve their function/For ease of cleaning/To reduce caries/Other reasons/I don't know

5 Has your child ever worn an orthodontic appliance or is your child wearing one now?

Yes/No/I don't know
